# Combining Postural Sway Parameters and Machine Learning to Assess Biomechanical Risk Associated with Load-Lifting Activities

**DOI:** 10.3390/diagnostics15010105

**Published:** 2025-01-04

**Authors:** Giuseppe Prisco, Maria Agnese Pirozzi, Antonella Santone, Mario Cesarelli, Fabrizio Esposito, Paolo Gargiulo, Francesco Amato, Leandro Donisi

**Affiliations:** 1 Department of Medicine and Health Sciences, University of Molise, 86100 Campobasso, Italy; g.prisco2@studenti.unimol.it (G.P.); antonella.santone@unimol.it (A.S.); 2 Department of Advanced Medical and Surgical Sciences, University of Campania Luigi Vanvitelli, 80138 Naples, Italy; mariaagnese.pirozzi@unicampania.it (M.A.P.); fabrizio.esposito@unicampania.it (F.E.); 3 Department of Engineering, University of Sannio, 82100 Benevento, Italy; mcesarelli@unisannio.it; 4 Institute of Biomedical and Neural Engineering, Reykjavik University, 102 Reykjavik, Iceland; paolo@ru.is; 5 Department of Information Technology and Electrical Engineering, University of Naples Federico II, 80125 Naples, Italy; framato@unina.it

**Keywords:** biomechanical risk assessment, machine learning, physical ergonomics, postural sway, Revised NIOSH Lifting Equation, wearable inertial sensors, weight lifting

## Abstract

**Background/Objectives**: Long-term work-related musculoskeletal disorders are predominantly influenced by factors such as the duration, intensity, and repetitive nature of load lifting. Although traditional ergonomic assessment tools can be effective, they are often challenging and complex to apply due to the absence of a streamlined, standardized framework. Recently, integrating wearable sensors with artificial intelligence has emerged as a promising approach to effectively monitor and mitigate biomechanical risks. This study aimed to evaluate the potential of machine learning models, trained on postural sway metrics derived from an inertial measurement unit (IMU) placed at the lumbar region, to classify risk levels associated with load lifting based on the Revised NIOSH Lifting Equation. **Methods**: To compute postural sway parameters, the IMU captured acceleration data in both anteroposterior and mediolateral directions, aligning closely with the body’s center of mass. Eight participants undertook two scenarios, each involving twenty consecutive lifting tasks. Eight machine learning classifiers were tested utilizing two validation strategies, with the Gradient Boost Tree algorithm achieving the highest accuracy and an Area under the ROC Curve of 91.2% and 94.5%, respectively. Additionally, feature importance analysis was conducted to identify the most influential sway parameters and directions. **Results**: The results indicate that the combination of sway metrics and the Gradient Boost model offers a feasible approach for predicting biomechanical risks in load lifting. **Conclusions**: Further studies with a broader participant pool and varied lifting conditions could enhance the applicability of this method in occupational ergonomics.

## 1. Introduction

The primary goal of ergonomics is to establish a working environment that is both safe and comfortable, enabling workers to carry out their tasks effectively while minimizing risks to their health [1,2,3]. Work-related musculoskeletal disorders (WRMDs) are injuries or conditions affecting the muscles, tendons, ligaments, nerves, and other soft tissues arising from the physical demands of the workplace. Research has shown a link between the incidence of WRMDs and occupational risk factors, including duration, intensity, and repetitive load lifting [4]. The European Agency for Safety and Health at Work reports that back-related issues account for approximately 43% to 46% of all WRMD cases [5].

Several quantitative or semi-quantitative methods are employed in occupational ergonomics to evaluate the biomechanical risk in developing WRMDs in various workstations. These methodologies, based on identifying repetitive movements and tasks, are valuable tools for identifying ergonomic risks [6,7,8,9,10,11,12]. Among current methods, the Revised National Institute for Occupational Safety and Health (NIOSH) lifting equation (RNLE) [13] is widely regarded as the most effective approach for assessing back pain risk associated with both single and repetitive lifting tasks in the workplace [14,15]. This model takes into account factors such as intensity, duration, frequency of the lifting, and the geometric characteristics of the load lifting to assess the biomechanical risk level. Despite its effectiveness, using such tools in ergonomic assessments can be labor-intensive and challenging, largely due to the absence of a standardized framework to streamline the process [16].

In response to this gap, wearable sensor technology has gained traction, driven by advancements in activity recognition and sensor applications in research. Wearable sensors—which allow measurement of inertial signals (linear acceleration and angular velocity), surface electromyography signals, and pressure signals—are increasingly being integrated into clinical settings. Their integration is particularly prominent within occupational health and physical ergonomics [17,18], where they play a pivotal role in monitoring work activities and preventing WRMDs. Moreover, the combined use of wearable sensors and artificial intelligence has yielded remarkable results in assessing biomechanical risk exposure, as highlighted in several scientific literature studies [19,20,21,22,23,24]. Donisi et al. [25] proposed a methodology to assess biomechanical risk according to the RNLE, using a Logistic Regression (LR) model trained with time- and frequency-domain features extracted from inertial signals acquired using a single Inertial Measurement Unit (IMU) placed on the sternum. This model achieved a classification accuracy of 82.8%. In an earlier study, the same authors [26] examined several machine learning models for classifying biomechanical risk levels, based on features extracted from inertial signals acquired using a single lumbar-mounted IMU. Their findings indicated that the Gradient Boost (GB) Tree model was the most effective, achieving a classification accuracy of 95%. Prisco et al. [27] explored the capability of machine learning algorithms to discriminate safe and unsafe lifting postures by utilizing time- and frequency-domain features extracted from inertial sensor data relating to the sternum. Their study found that LR was the most effective classifier, achieving an accuracy of 96%. Conforti et al. [28] focused on posture assessment, specifically distinguishing correct and incorrect body positions during lifting tasks using a Support Vector Machine (SVM) model fed with kinematic data from eight IMUs, reaching an accuracy of 76.9%. Alwasel et al. [29] used an SVM model combined with a motion capture suit fitted with 17 IMUs to classify poses in masonry work, achieving a classification accuracy of 92%. In another study, Chen et al. [30] implemented a multi-class SVM approach based on motion tensor decomposition with 17 IMUs to identify awkward postures, achieving an accuracy of up to 99%. Thomas et al. [31] applied a machine learning framework based on a modified Deep Convolutional and LSTM Recurrent Neural Networks (DeepConvLSTM) architecture, utilizing six IMUs throughout the body to classify risky liftings according to the American Conference of Governmental Industrial Hygienists (ACGIH) Threshold Limit Values (TLV). This model achieved an accuracy of 96%. Similarly, Snyder et al. [32] developed a classification method based on the ACGIH TLV guidelines, using a 2D convolutional neural network (CNN) with six body-mounted IMUs, achieving an accuracy of 90.6%.

Although substantial progress has been achieved in recent years, to the best of our knowledge, there is still a lack of research studies integrating postural sway measurements into machine learning models to evaluate biomechanical risk during liftings. Indeed, lifting loads while managing postural instability poses a significant challenge to the human body, especially when factors such as the geometric characteristics, weight, and frequency of the lifts are variable. Such instability can lead to a rapid onset of both muscular and physiological fatigue, heightening the risk of injury for workers [33,34]. As the weight of a load increases, the center of gravity of the body shifts, demanding constant postural adjustments. These adjustments rely heavily on stabilizing muscles, particularly those along the spinal column, which play a crucial role in maintaining balance and posture [35]. Over time, this increased muscular effort accelerates fatigue and raises the likelihood of errors in posture, which, in turn, elevates the risk of WRMDs. Additionally, both the load’s weight and the frequency of lifting contribute to the development of fatigue. Heavier weights place greater strain on muscles, while frequent repetitions reduce recovery periods, causing fatigue to build up and impairing motor control [36,37]. Only one study, D’Anna et al. [38], investigated the potential of using postural sway measurements to categorize risk levels associated with repetitive lifting activities based on the RNLE.

Given the existing literature and the gap in studies utilizing IMU-derived postural data for biomechanical risk classification, this work aimed to assess the feasibility of machine learning algorithms fed with postural sway parameters to classify biomechanical risk levels during lifting tasks using a single IMU placed on the lumbar region, aligning with the body’s center of mass (COM). In addition, the work aimed to identify the most informative sway parameters and the primary direction of sway to better understand how postural control adapts as biomechanical risk increases.

## 2. Materials and Methods

### 2.1. Wearable Inertial System

**Figure 1:**
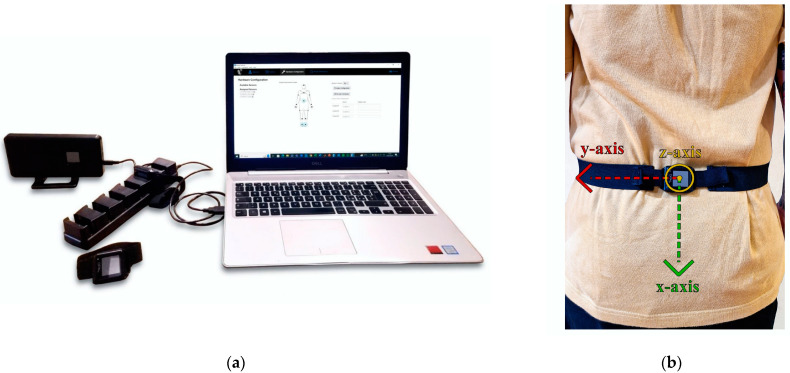
(**a**) Mobility Lab System: access point, opal sensors, docking station and Moveo Explorer software. (**b**) opal sensor placement and local coordinate frame.

The Mobility Lab system (APDM Inc., Portland, OR, USA) is a portable wearable inertial system tailored for clinicians and researchers focused on human movement analysis. This system includes several components: (1) set of opal inertial sensors worn on the body and connected wirelessly, (2) docking station used to charge and configure these sensors, (3) access point for synchronizing data transmission from the sensors via Bluetooth 3.0, and (4) Moveo Explorer software (version 1.0.0.202206161718) that simplifies protocol setup and provides automated data analysis and reporting (Figure 1a).

The opal sensors, based on IMUs, record linear acceleration and angular velocity signals at a sampling frequency of 128 Hz. Each sensor includes a tri-axial accelerometer with 14-bit resolution, a 50 Hz bandwidth, and a range of ±200 g; a tri-axial gyroscope with 16-bit resolution, a 50 Hz bandwidth, and a range of ±2000 deg/s; and a tri-axial magnetometer with 12-bit resolution, a 32.5 Hz bandwidth, and a range of ±8 Gauss. The software offers instrumented versions of standard protocols for detailed quantitative data analysis in addition to offering the possibility of extracting the raw signals associated with the movement. In the present study, a single opal sensor placed on the lumbar region was used, as reported in Figure 1b. The Mobility Lab System has been proven to be repeatable and accurate [39,40].

### 2.2. Revised NIOSH Lifting Equation

The RNLE was used to assess the biomechanical risk levels. This consists of a mathematical model incorporating seven task-related variables allowing the calculation of the Recommended Weight Limit (RWL) [14], as shown in Equation (1):

RWL = LC × HM × VM × DM × AM × FM × CM(1)

where:

- Load Constant (LC): the maximum load nearly all healthy workers should be able to lift under optimal conditions;
- Horizontal Multiplier (HM): depends on the horizontal distance from the midpoint between the ankles and hands while holding the object;
- Vertical Multiplier (VM): depends on the vertical distance of the hands from the ground at the beginning of lifting;
- Distance Multiplier (DM): depends on the vertical distance travelled by the load;
- Asymmetric Multiplier (AM): depends on the twisting angle of the body during lift;
- Frequency Multiplier (FM): depends on the frequency of lifting in a shift;
- Coupling multiplier (CM): depends on the quality of the grip classified as good, fair, or poor.

The RWL represents the maximum load that healthy workers can lift without the risk of developing lower back injuries over a sustained period. After determining the RWL, the Lifting Index (LI) can be calculated by dividing the load lifted by the RWL [41,42]. LI offers a relative measure of the physical strain and the potential risk of developing WRMDs associated with the lifting task. An LI value below 1 indicates an acceptable level of risk, whereas an LI greater than 1 suggests an increased likelihood of WRMDs.

### 2.3. Study Population

In the present study, 8 subjects (3 males and 5 females) in the age range of 20–30 years were enrolled. All participants were required to meet the following inclusion criteria: (1) no musculoskeletal disorders or other occupational pathologies and (2) within the working age. The study was approved by the local Ethics Committee in accordance with the Declaration of Helsinki, and all the participants signed a declaration of informed consent. The demographic characteristics are summarized in Table 1.

### 2.4. Experimental Protocol

Participants completed two sessions of load-lifting tasks, each corresponding to a specific risk level according to RNLE. The first session was based on a LI < 1 (0.5), categorizing it as the “No-Risk task”, while the second session, based on a LI > 1 (1.3), was associated with the “Risk task”. In each session, participants performed 20 lifts with a frequency defined as reported in Table 2. To determine the LI using the RNLE, various multipliers were applied as outlined in the NIOSH lifting manual [14]. Calculations of the LC, HM, VM, and DM values followed the specified formulas in [14]. Since no twisting was required, the AM value was set to 1. Additionally, because the crate had handles, making it easier to lift, the CM was set to 1 (good). Details of the lifting tasks are provided in Table 2.

### 2.5. Digital Signal Processing and Feature Extraction

**Figure 2:**
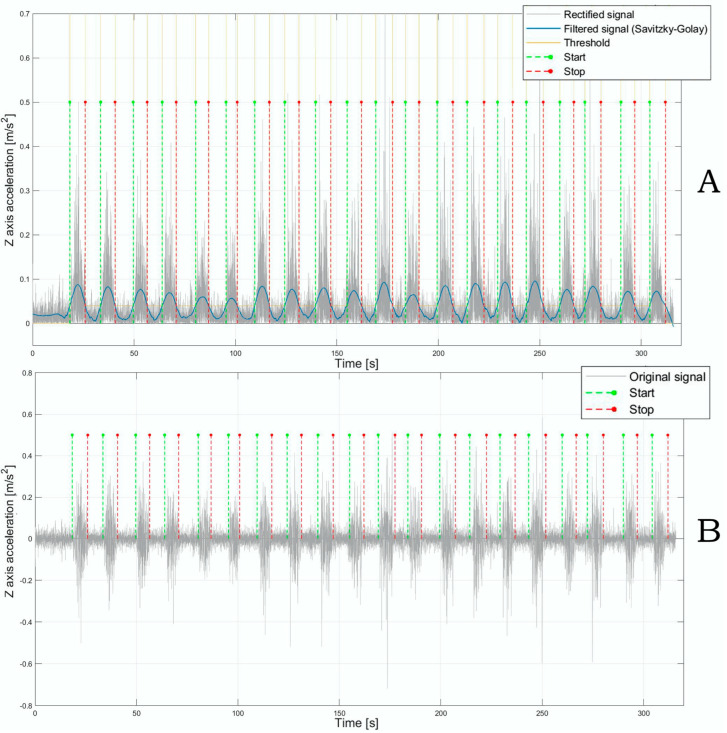
(**A**) The rectified signal in grey, and the rectified and filtered signal via the Savitsky-Golay filter in blue, the threshold in yellow, and the start and stop points in green and red, respectively. (**B**) The original signal in grey, with start and stop points in green and red, respectively.

Acceleration data were collected throughout the lifting activities, concentrating on the z-axis and y-axis, which represent the anteroposterior (AP) and mediolateral (ML) directions, respectively. The data were segmented to detect specific regions of interest (ROIs), focusing on segments that corresponded to the lifting motions. The segmentation process was initially triggered by the z-axis acceleration signal, as this axis showed the most significant amplitude variations during lifting. Subsequently, the time windows corresponding to the lifting actions obtained from the segmentation process were applied both to the z- and y-axis acceleration data. To preserve the spectral content of the signal and eliminate the offset, a 4th order Butterworth band-pass filter was applied to the signal, restricting frequencies to a range of 1–50 Hz. Next, the signal was first rectified and then underwent additional smoothing through a 2nd order Savitzky–Golay filter with a frame length of 1501. For each subject, a custom threshold was established through an iterative approach, and the start and end points of each ROI were identified by locating where the threshold intersected with the processed signal, as shown in Figure 2A. Finally, the start and stop points were then applied to the signal, which was first processed using a 4th-order Butterworth band-pass filter (1–50 Hz) (Figure 2B).

**Figure 3:**
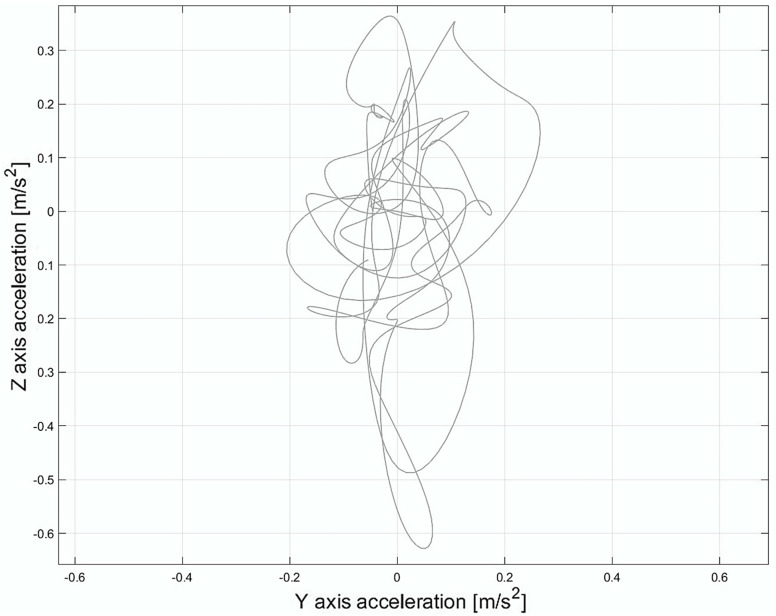
Stabilogram in the acceleration domain was derived by using an inertial sensor placed on the lumbar region, considering the anteroposterior direction (z-axis) and mediolateral direction (y-axis) within a single ROI.

The z- and y-axis acceleration signals were down-sampled to 20 Hz and filtered with a 3rd order Butterworth low-pass filter, setting the cut-off frequency at 5 Hz to extract postural sway parameters. The mean value was then subtracted, following the method suggested by Agostini et al. [43]. Figure 3 illustrates the resulting postural sway stabilogram in the acceleration domain, derived from the acceleration data along the AP and ML axes within one specific ROI.

Ten time-domain and frequency-domain sway parameters were computed from each acceleration stabilogram in a specific ROI, according to the formulas reported in [44]. The following time-domain features were extracted:

- Mean distance (MDIST) (m/s^2^) represents the average distance from the mean COM:

(2) MDIST=1N∑n=1NRD(n)

where RD(n) is:

(3) $$RD\left(n\right)={\left[{AP(n)}^{2}+{ML(n)}^{2}\right]}^{1/2}$$

- Mean distance AP (MDIST_AP_) (m/s^2^) represents the average AP distance from the mean COM:

(4) MDISTAP=1N∑n=1NAP(n)

- Mean distance ML (MDIST_ML_) (m/s^2^) represents the average ML distance from the mean COM:

(5) MDISTML=1N∑n=1NML(n)

- Range of distance (RDIST) (m/s^2^) is the maximum distance between any two points on the COM path:

(6) $$RDIST=\left|\max\left(RD\right)-min(RD)\right|$$

- Range of distance AP (RDIST_AP_) (m/s^2^) is the maximum AP distance between any two points on the COM path:

(7) $${RDIST}_{AP}=\left|\max\left(AP\right)-min(AP)\right|$$

- Range of distance ML (RDIST_ML_) (m/s^2^) is the maximum ML distance between any two points on the COM path:

(8) $${RDIST}_{ML}=\left|\max\left(ML\right)-min(ML)\right|$$

- Root mean square of distance (RMS-DIST) (m/s^2^) is the Root mean square (RMS) value of the resultant distance (RD) time series:

(9) RMS−DIST=1N∑n=1NRD(n)21/2

- RMS of distance AP (RMS-DIST_AP_) (m/s^2^) is the Root mean square (RMS) value of the AP time series:

(10) RMS−DISTAP=1N∑n=1NAP(n)21/2

- RMS of distance ML (RMS-DIST_ML_) (m/s^2^) is the Root mean square (RMS) value of the ML time series:

(11) RMS−DISTML=1N∑n=1NML(n)21/2

- Mean velocity (MV) (m/s) is the average velocity of the COM:

(12) MV=TOTEXT

where TOTEX is:

(13) $$TOTEX=\sum_{n=1}^{N-1}{\left[{\left(AP\left(n+1\right)-AP(n)\right)}^{2}+{\left(ML\left(n+1\right)-ML(n)\right)}^{2}\right]}^{1/2}$$

- Sway Area (SA) (m^2^/s^3^) estimates the area enclosed by the COM path per unit of time:

(14) $$SA=\frac{1}{2T}\sum_{n=1}^{N-1}\left|AP\left(n+1\right)ML\left(n\right)-AP(n)ML(n+1)\right|$$

Frequency-domain features were chosen for their capacity to characterize the area or shape of the power spectral density, namely G(m), which was calculated using the Fast Fourier Transform (FFT) algorithm. The following sway features in the frequency domain were then extracted:

- Mean frequency (MF) (Hz) is the rotational frequency of the COM if it had traveled the total excursions around a circle with a radius of the mean distance:

(15) $$MF=\frac{TOTEX}{2\pi MD\cdot T}$$

- Centroidal frequency (CF) (Hz) is the frequency at which the spectral mass is concentrated:

(16) CF=μ2μ01/2

where $\mu_{k}$ is:

(17) $$\mu_{k}=\sum_{m=1}^{N}{\left(m∆f\right)}^{k}G(m)$$

- Dispersion frequency (DF) (adim) is a unitless measure of the variability in the frequency content of the power spectral density:

(18) DF=1−μ12μ0μ21/2

where:

- AP(n) is the nth sample of AP acceleration signal within a single ROI;
- ML(n) is the nth sample of ML acceleration signal within a single ROI;
- N is the number of samples of signal within a single ROI;
- RD is resultant distance;
- TOTEX is the total length of the COM path;
- T is the period of time of signal within a single ROI;
- m is mth sample of signal in frequency-domain;
- G(m) is the discrete power spectral density of RD;
- Δf is the frequency increment of G(m);
- μ_k_ is kth spectral moment.

Although the original formulas were developed for Center of Pressure (COP) signals, as referenced in [44], the present study extends their application to the analysis of acceleration signals associated with the COM. This methodology is supported by the scientific literature, which demonstrates a significant correlation between postural metrics derived from lumbar accelerometer data and COP measurements [43,45].

### 2.6. Machine Learning Algorithms and Tools

The following nine supervised machine learning models were employed in the present study: SVM with polynomial kernel [46]; Decision tree (DT) [47]; GB [48]; Random Forest (RaF) [49]; Rotation Forest (RoF) [50]; eXtreme Gradient Boost (XGB) [51]; K-nearest neighbors (kNN) [52]; Multilayer Perceptron (MLP) [53]; Probabilistic Neural Network (PNN) [54].

As a validation strategy, a stratified k-fold Cross-Validation (CV) was employed [55]. Stratified k-fold CV splits the dataset into k equal parts, called folds, ensuring that the distribution of the target variable is preserved in each fold. In each iteration, the model is trained on k-1 folds while the remaining fold is used for testing. This cycle continues k times, ensuring every fold is utilized once as a test set. The final performance score is calculated by averaging the results from all k trials, offering a more reliable measure of the model’s accuracy. In the current study, k was set equal to 10.

As a feature selection technique, Backward Feature Elimination (BFE) was used [56] in order to improve model performance and interpretability. It starts with all features, removing the least significant ones based on performance loss. This process continues until only the most relevant features remain, reducing overfitting and balancing accuracy with model simplicity. In the current study, the machine learning model, which was subsequently employed for training and testing, was utilized. This approach ensures that the feature selection process is aligned with the model’s specific structure and performance characteristics [57].

Furthermore, hyperparameter optimization for all models was carried out using a Bayesian approach. To facilitate reproducibility, the optimized hyperparameters are detailed as follows. For SVM, the Bias, Gamma, and Power values were set to 1.033, 1.971, and 1.99, respectively. In the DT model, the minimum record per node was set to 6, the total record reviewed during training to 5290, and the maximum distinct nominal values per feature to 3. For GB, the maximum depth of each tree (maxLevels) was set to 7, with 93 trees (nrModels) and a learning rate of 0.186. In RaF, the number of trees (nrModels) was set to 57, the maximum tree depth (maxLevels) to 20, and the minimum node size (minNodeSize) to 7. In XGB, the maximum leaves per tree (maxLeaves) was set to 21, the learning rate (eta) to 0.212, with gamma at 0, lambda at 100, and a maximum of 59 bins (maxBins). For kNN, the value of k was set to 3. For MLP, the maximum number of iterations was set to 109, with 2 hidden layers and 43 neurons per layer. For PNN, theta minus, theta plus, and maximum of epochs were set equal to 0.113, 0.509, and 131, respectively. Finally, for RoF, default parameters were used.

In addition, for the kNN, MLP, and SVM models, a max-min normalization technique was applied to rescale feature values between 0 and 1. This approach ensures that all features contribute comparably to the model, preventing any one feature from dominating due to differences in scale [58].

The following evaluation metrics were calculated to assess the performances of the implemented machine learning classifiers: accuracy, F-measure, specificity, sensitivity, precision, recall, and area under the receiver operating curve (AUCROC) [59,60]. AUCROC scores indicate the machine learning models’ effectiveness in distinguishing between the different classes. These scores are generally categorized into three levels: moderate (0.70–0.80), good (0.80–0.90), and excellent discrimination ability (above 0.90).

Additionally, to identify the most discriminative sway parameters, a feature importance analysis was conducted using the Information Gain (IG) method [61].

KNIME Analytics Platform (version 4.1.3), a platform widely used in several research articles about physical ergonomics [62,63], was used for machine learning analysis.

## 3. Results

**Figure 4:**
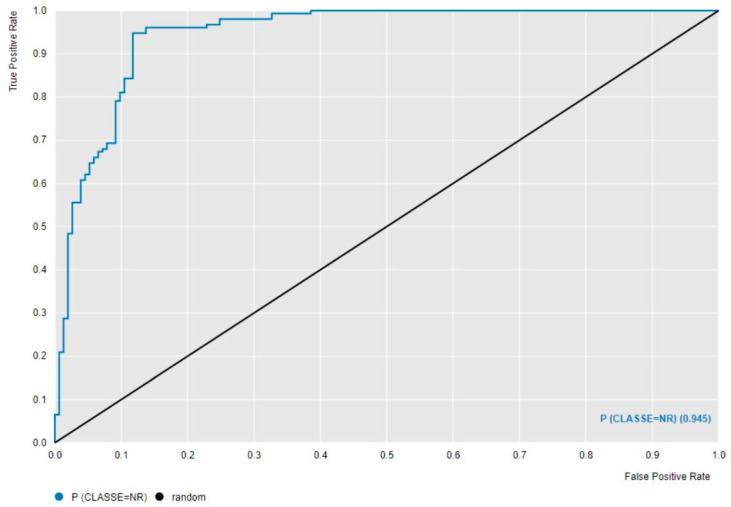
ROC curve of GB, which was found to be the best machine learning classifier in the biomechanical risk assessment associated with load-lifting activities; NR: No-Risk.

First, machine learning analysis was performed to assess whether the combination of the implemented machine learning classifiers—leveraging postural sway parameters derived from AP and ML linear acceleration signals of the lumbar region—along with feature selection and hyperparameter optimization techniques, could accurately classify the presence of biomechanical risk during load-lifting tasks. The performance metrics for all machine learning algorithms were reported in Table 3. The corresponding confusion matrix and AUCROC of the best classifier GB are shown in Table 4 and Figure 4, respectively.

**Figure 5:**
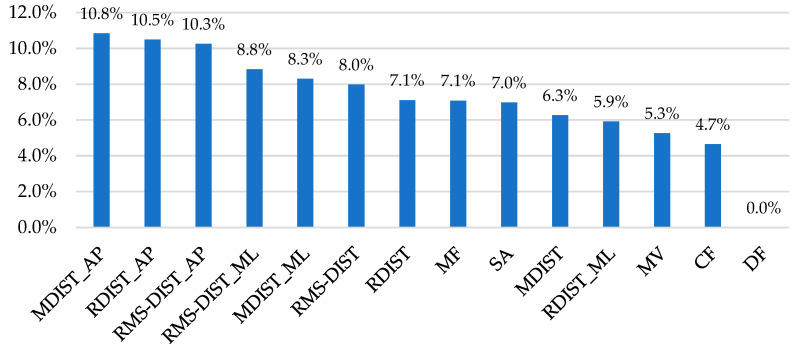
Ranking of the postural sway features based on IG methods. The abbreviations are defined as follows:: MDIST_AP = Mean distance along anteroposterior direction; RDIST_AP = Range of distance along anteroposterior direction; RMS-DIST_AP = Root mean square of distance along anteroposterior direction; RMS-DIST_ML = Root mean square of distance along mediolateral direction; MDIST_ML = Mean distance along mediolateral direction; RMS-DIST = Root mean square of distance; RDIST = Range of distance; MF = Mean frequency; SA = Sway Area; MDIST = Mean distance; RDIST_ML = Range of distance along mediolateral direction; MV = Mean velocity; CF = Centroidal frequency; DF = Dispersion frequency.

Second, a feature importance analysis—based on the IG method—was performed to identify the features most important for predicting the two classes. In Figure 5, the ranking of each feature according to its importance score was reported.

## 4. Discussion

The present study focused on evaluating the potential of machine learning techniques to classify biomechanical risk levels during lifting activities using postural sway metrics obtained from a single IMU positioned on the lumbar region, closely aligned with the body’s COM. Furthermore, it sought to determine the most relevant sway parameters and the dominant direction of postural adjustments, providing insights into how balance control evolves under increasing biomechanical strain.

Table 3 presents the evaluation metrics for all machine learning models coupled with BFE and hyperparameters optimization techniques. Most models achieved performance ranging from good to excellent, with overall accuracy exceeding 80%, except for PNN, which attained an accuracy of 69.6%. The lower performance of the PNN algorithm may be likely attributable to correlations among the sway features, as probabilistic classifiers typically assume feature independence—a condition that is not fully satisfied in this case. GB emerged as the top-performing machine learning classifier, achieving an accuracy of 91.2%, along with F-measure, specificity, sensitivity, precision, recall, and AUCROC values of 91.5%, 87.6%, 94.8%, 8.84%, 94.8%, and 94.5%, respectively. GB successfully classified 134 instances out of 306, as detailed in Table 4. Additionally, GB achieved an AUCROC score of 94.5%, demonstrating an outstanding capability to differentiate the No-Risk and Risk classes (Figure 4).

To evaluate the role of postural sway features in distinguishing between No-Risk and Risk classes, a feature importance analysis was performed using the IG method. As illustrated in Figure 5, all sway metrics except DF had non-zero ranking values, highlighting their relevance in predicting biomechanical risk exposure. Among these, MDIST_AP_ was the most predictive feature (10.8%), followed closely by RDIST_AP_ (10.5%) and RMS-DIST_AP_ (10.3%). Other notable features included RMS-DIST_ML_ (8.8%), MDIST_ML_ (8.3%), RMS-DIST (8.0%), RDIST (7.1%), MF (7.1%), SA (7.0%), MDIST (6.5%), RDIST_ML_ (5.9%), MV (5.3%), and CF (4.7%).

**Figure 6:**
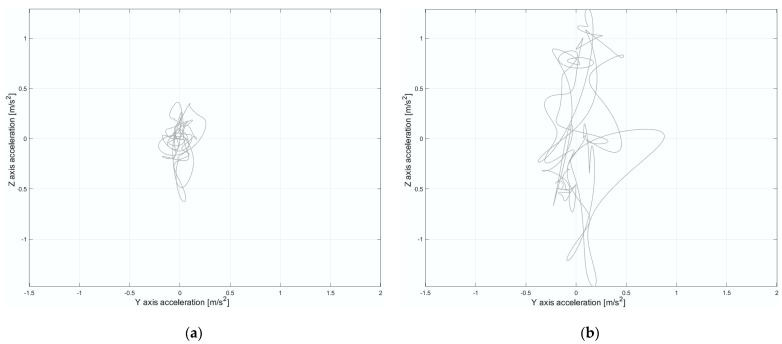
(**a**) Stabilogram in the acceleration domain considering the anteroposterior direction (z-axis) and mediolateral direction (y-axis) within a single ROI associated with the No-Risk class. (**b**) Stabilogram in the acceleration domain considering the anteroposterior direction (z-axis) and mediolateral direction (y-axis) within a single ROI associated with the Risk class.

This analysis revealed that time-domain parameters were more predictive than frequency-domain parameters, accounting for 88.3% of the total ranking value. This finding emphasizes the potential to reduce computational demands by focusing solely on time domain characteristics in a way that facilitates real-time assessments. However, further investigation is needed to assess how this approach might affect the predictive performance of machine learning models. Moreover, the AP direction emerged as the most predictive for postural sway during load lifting, with a ranking value of 57.8%. This suggests that as the weight increases in the Risk class, the overall COM of the system (subject + load) shifts further away from the base of support (the area bounded by the feet). Consequently, imbalance increases with heavier loads, requiring greater muscle effort and more precise postural control to maintain stability. Additionally, in the current study, the absence of an asymmetry factor (twisting angle of the body during lifting set to zero) suggests that the most significant imbalance occurs specifically along the AP direction, as illustrated in Figure 6. This observation underscores the critical role of postural sway features in biomechanical risk assessment.

Several studies have investigated the classification of risk levels during lifting tasks using machine learning algorithms. For example, Donisi et al. [25] proposed an automated method for assessing biomechanical risk based on the RNLE. Their approach employed an LR model trained on time- and frequency-domain features extracted from data collected via a single IMU sensor positioned on the sternum, achieving an accuracy of 82.8%. However, as the authors acknowledged, relying solely on a statistical model introduced significant limitations in their study. In contrast, the present study evaluated multiple machine learning models, achieving a considerably higher accuracy of 91.2%. In another study [26], the same authors investigated the performance of various machine learning algorithms under similar conditions, this time using a single IMU mounted on the lumbar region. Similarly to the present study, they identified the GB model as the best classifier, attaining an accuracy of 95%. Unlike their approach, this study placed deliberate emphasis on sway parameters, aiming not only to classify lifting tasks but also to explore how overall body balance affects biomechanical risk during lifting. Thus, providing a more comprehensive understanding of the relationship between postural control and risk classification.

Alwasel et al. [29] and Chen et al. [30] explored the use of SVM models leveraging joint kinematic data collected from 17 IMUs placed across the body to identify awkward postures. Alwasel et al. [29] reported an accuracy of 92.0%, while Chen et al. [30] achieved an accuracy of up to 99%. Similarly, Snyder et al. [32] and Thomas et al. [31] proposed methods to assess biomechanical risks during lifting activities based on the ACGIH TLV guidelines. Snyder et al. [32] employed a 2D CNN trained on kinematic data from 6 IMUs placed on the upper back, wrists, dominant upper arm, waist, and dominant thigh, achieving 90.6% accuracy. Thomas et al. [31] utilized a modified DeepConvLSTM framework under similar conditions, yielding an accuracy of 96%. While these methods demonstrated strong predictive performance, they face practical challenges in real-world applications due to their reliance on multiple sensors. As the authors themselves noted, these challenges include higher costs and increased complexity in sensor placement, making such setups more suited for controlled laboratory environments. In contrast, this study emphasizes the advantages of reducing the number of sensors, minimizing redundancy and complexity while still achieving high accuracy. Therefore, the approach developed by the authors offers a more feasible solution for real-world applications. Finally, D’Anna et al. [38] investigated the role of postural parameters extracted from the COP in assessing risk levels associated with repetitive lifting activities, as defined by the RNLE. Additionally, they examined whether changes in COP parameters correlate with established surface electromyography (sEMG) measures of muscle activity, commonly used as indicators of muscular fatigue. The study involved 14 healthy control participants, each of whom performed 3 lifting sessions at 3 different risk levels based on the RNLE criteria. Postural sway parameters were extracted from the COP trajectories recorded via a force platform. Statistical analyses were conducted to determine whether these postural sway parameters were significantly effective in discriminating between risk levels. Consistent with the findings of the presented study, the authors observed that all parameters, specifically COP velocity parameters in both the AP and mediolateral ML directions, increased significantly under high-risk conditions. This indicates the sensitivity of COP-derived parameters to variations in risk levels. Furthermore, significant correlations between sEMG parameters and COP velocity were identified, suggesting that muscular fatigue contributes to alterations in postural control, particularly under high LI conditions. These results align with the conclusions identified in this study. However, while their findings are promising, the study by D’Anna et al. [38] was limited to statistical analyses for evaluating the feasibility of categorizing risk levels. It did not employ machine learning techniques to assess the practical applicability of these findings. In contrast, the present study introduced a semi-automatic approach, utilizing GB models combined with postural sway parameters to classify biomechanical risk levels during weight-lifting activities. This approach underscores the potential for integrating sway metrics into occupational ergonomics, highlighting their utility in real-world applications.

Based on these findings, the proposed methodology, which combines the sway parameters and machine learning algorithms, provides a valid approach to distinguish biomechanical risk classes (No-Risk/Risk) during manual load lifting in order to prevent WRMDs. Furthermore, the study highlights that incorporating postural sway parameters, which capture dynamic shifts in balance, provides a more comprehensive understanding of biomechanical stresses, particularly under varying working conditions. Consequently, the combined use of sway parameters and machine learning algorithms significantly enhances the accuracy of biomechanical risk assessments.

## 5. Conclusions

This study presents an innovative approach that combines machine learning models with postural sway parameters to classify biomechanical risk levels during weightlifting tasks, as defined by the RNLE. The sway parameters, derived from linear acceleration data that describe the COM movement in the transverse plane, effectively quantify biomechanical risk exposure. This approach enhances existing protocols by offering a more efficient and accessible way to assess biomechanical risks and provides an alternative when standardized evaluation methods cannot be applied. Unlike previous studies, this approach focuses on the dynamic aspects of postural control, offering deeper insights into how balance mechanisms adapt to biomechanical strain. Notably, the present study highlights the predominance of AP sway metrics as pivotal indicators of biomechanical stress, further solidifying their role in biomechanical risk assessment. This innovative methodology has the potential to complement and enhance well-established approaches in occupational ergonomics for evaluating biomechanical risks.

Nonetheless study limitations include the small sample size and demographic imbalances among subjects that should be addressed in future research. An additional limitation of this preliminary study lies in the simplified protocol, which includes only two biomechanical risk classes. Moreover, only weight lifted and lifting frequency were varied between the two biomechanical risk classes. Therefore, it will be necessary to evaluate the proposed approach on more general scenarios, and then future studies should consider additional biomechanical risk classes, also allowing a greater variability in load-lifting parameters to improve the robustness and generalizability of these findings. Finally, larger datasets are mandatory to verify the robustness and generalizability of the ML algorithms to classify biomechanical risk classes. Moreover, the proposed methodology should be assessed separately for male and female participants to facilitate a comprehensive understanding of its performance across genders.

Future research should aim to extend the proposed methodology to additional manual work tasks beyond lifting and real-time evaluations, enhancing its practical utility in the occupational ergonomic field. Moreover, the proposed methodology should be assessed separately for male and female participants to facilitate a comprehensive understanding of its performance across genders. Finally, it could be useful to test the impact of using other acceleration signals (i.e., x-axis or y-axis acceleration) and/or other approaches (i.e., automatic or manual) to segment the signals and to extract the ROIs relating to the weight-lifting and evaluate the robustness of the achieved results.

## Figures and Tables

**Table 1:** Demographic characteristics of the study population were reported as mean ± standard deviation.

Characteristics	Mean ± Standard Deviation
Age (years)	27.29 ± 1.70
Height (cm)	171.71 ± 8.83
Weight (kg)	63.71 ± 12.81
Body Mass Index (kg/m^2^)	21.41 ± 2.62

**Table 2:** The details of the lifting tasks corresponding to LI < 1 and LI > 1 according to the RNLE.

Trial 1 (LI < 1, LI = 0.5)				Trial 2 (LI > 1, LI = 1.3)				
Vertical Displacement [cm]	Frequency [lifts/min]	Weight Lifted [kg]		Vertical Displacement [cm]	Frequency [lifts/min]		Weight Lifted [kg]	
M and F	M and F	M	F	M and F	M	F	M	F
50–120	2.5	7	5	50–120	6	4	15	10

Abbreviations: M: male, F: female.

**Table 3:** Evaluation metric scores of machine learning algorithms using stratified 10-fold CV, BFE and hyperparameters optimization.

	SVM	DT	GB	RaF	XGB	RoF	kNN	MLP	PNN
**Accuracy**	0.843	0.886	0.912	0.889	0.856	0.859	0.879	0.873	0.696
**F-measure**	0.861	0.887	0.915	0.896	0.862	0.867	0.882	0.877	0.59
**Specificity**	0.712	0.876	0.876	0.824	0.810	0.804	0.856	0.837	0.954
**Sensitivity**	0.974	0.895	0.948	0.954	0.902	0.915	0.902	0.908	0.438
**Precision**	0.772	0.878	0.884	0.844	0.826	0.824	0.863	0.848	0.905
**Recall**	0.974	0.895	0.948	0.954	0.902	0.915	0.902	0.908	0.438
**AUCROC**	0.926	0.918	0.945	0.953	0.940	0.934	0.999	0.939	0.929

**Table 4:** Confusion matrix of GB as the best machine learning classifier.

	NR	R
**NR**	145	8
**R**	19	134

Abbreviations: NR = No-Risk class, R = Risk class.

## Data Availability

The datasets generated and analyzed in this study are not publicly available due to the privacy policy but are available from the corresponding author upon reasonable request.
